# MicroRNA-30b-5p promotes the proliferation and migration of human airway smooth muscle cells induced by platelet-derived growth factor by targeting phosphatase and tensin homolog deleted on chromosome ten

**DOI:** 10.1080/21655979.2021.1950401

**Published:** 2021-07-12

**Authors:** Wentao Wang, Jian Guo, Yan Wang

**Affiliations:** a Department of Pediatrics, Affiliated Hospital of Chengde Medical University, Chengde City, Hebei Province, China; b Department of Neonatology, Affiliated Hospital of Chengde Medical University, Chengde City, Hebei Province, China

**Keywords:** Airway smooth muscle cell, asthma, miR-30b-5p, PTEN, cell proliferation, cell migration

## Abstract

Dysfunction of airway smooth muscle (ASM) cells is crucial in asthma pathogenesis. Here, microRNA-30b-5p (miR-30b-5p)’s function and mechanism in ASM cells’ multiplication and migration were investigated. Microarray was utilized for identifying the differentially expressed miRNAs in the bronchial epithelial cells of the asthma patients and healthy controls. Platelet-derived growth factor (PDGF) was employed to treat ASM cells to establish an *in-vitro* asthma model. Quantitative real-time PCR (qRT-PCR) was conducted for detecting the expressions of miR-30b-5p and phosphatase and tensin homolog deleted on chromosome 10 (PTEN). 3-(4,5-Dimethylthiazol-2-yl)-2,5-diphenyltetrazolium bromide (MTT) and 5-bromo-2ʹ-deoxyuridine (BrdU) assays were used for examining cell multiplication; Transwell assay was performed for detecting cell migration; cell cycle was analyzed through flow cytometry. The targeted relationship between PTEN and miR-30b-5p was verified using a dual-luciferase reporter gene assay. Western blot was used for detecting the expressions of phosphorylated (p)-phosphatidylinositol 3-kinase (PI3K), PTEN, PI3K, protein kinase B (AKT) and p-AKT in ASM cells. We demonstrated that, miR-30b-5p expression in the bronchial epithelial cells of asthmatic patients was up-regulated. It was also increased in PDGF-stimulated ASM cells. Transfection of miR-30b-5p mimics facilitated ASM cells’ multiplication, migration and cycle progression, while inhibiting miR-30b-5p had the opposite effect. Furthermore, miR-30b-5p could target PTEN to repress PTEN expression. PTEN overexpression attenuated the effect of miR-30b-5p on ASM cells. Moreover, miR-30b-5p overexpression facilitated the expression of p-PI3K and p-AKT in PDGF-stimulated ASM cells. Collectively, miR-30b-5p activates the PI3K/AKT pathway by targeting PTEN to facilitate PDGF-induced dysfunction of ASM cells.

## Graphical abstract

**Figure:**
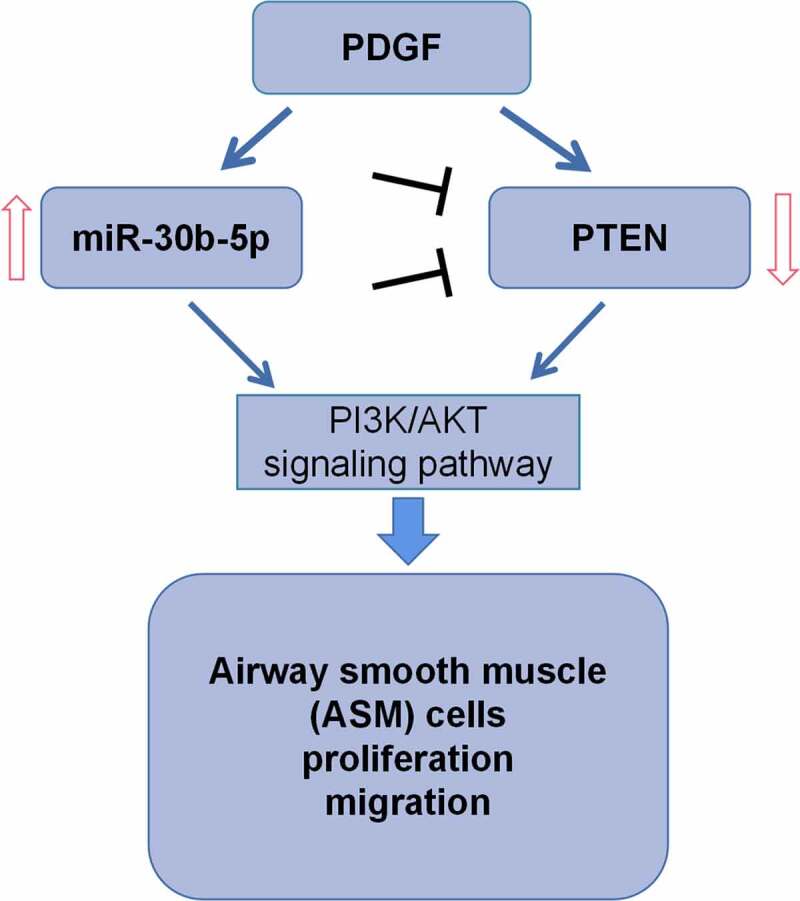

## 1. Introduction

Asthma is a chronic airway inflammatory disease, characterized by airflow obstruction, airway inflammation and airway remodeling [1]. Compared with healthy people, asthma patients have a higher incidence of pulmonary dysfunction, which is related to the progressive remodeling of the airway [2]. The main characteristics of this pathological process include epithelial injury, airway smooth muscle (ASM) cells’ proliferation and migration, glandular hyperplasia, and airway wall fibrosis [3,4]. Platelet-derived growth factor (PDGF) is significantly highly expressed in the lung tissues of asthmatic patients, and it takes part in promoting ASM cell multiplication and migration [5]. Therefore, elucidating the mechanism of PDGF-induced ASM cell multiplication and migration is of great significance for deciphering the pathogenesis of asthma.

MicroRNAs (miRs or miRNAs) are a class of non-coding RNA characterized by a length of 18–25 nucleotides [6]. MiRNAs bind to target mRNAs’ 3ʹ-untranslated region (3ʹUTR) to induce their degradation or translation inhibition, thereby regulating the stability of mRNA and protein expression at the post-transcriptional level [6]. A lot of miRNAs have been reported to be linked to the occurrence and development of multiple human diseases [7–12]. For instance, miR-548b-3p represses the progression of lung cancer [7]; miR-1269b represses the progression of gastric cancer development via regulating methyltransferase-like 3 (METTL3) [8]. MiR-30b-5p has been confirmed to have a tumor-suppressive effect in various cancers (including esophageal squamous cell carcinoma, glioma, hepatocellular carcinoma, etc.) [9–11]. Moreover, miR-30b-5p can promote the apoptosis of cardiomyocytes in rats with myocardial infarction via modulating the Wnt/β-catenin signal pathway [12]. MiR-30b-5p is up-regulated in AC16 cells under hypoxia; miR-30b-5p inhibition can alleviate hypoxia-induced injury of cardiomyocyte via targeting Aven [13]. In this study, bioinformatics analysis showed that in the bronchial epithelial cells of asthmatic patients, miR-30b-5p was abnormally highly expressed. Nevertheless, its functions and mechanisms in the asthma pathogenesis have not yet been elucidated.

Phosphatase and tensin homolog deleted on chromosome ten (PTEN) is a tumor suppressor with protein phosphatase and lipid phosphatase activity [14]. With the lipid phosphatase activity, PTEN can convert phosphatidylinositol [3–5]-triphosphoric acid (PIP3) into phosphatidylinositol 4,5-bisphosphate (PIP2) and antagonizes the PI3K-Akt-mTOR pathway, thus suppressing cell survival, proliferation and migration [14]. In recent years, PTEN’s role in airway inflammation and remodeling has received increasing attention. It is reported that that PTEN depletion contributes to promoting ASM cells’ proliferation and thus aggravating airway remodeling in asthma [15].

The molecular mechanism of ASM cells’ dysfunction is not clear. In the present work, we hypothesize miR-30b-5p is a crucial regulator for the phenotypes of ASM cells. The present study reports that PTEN is one of miR-30b-5p’s target genes, and miR-30b-5p can promote the dysfunction of ASM cells by repressing PTEN. To our best knowledge, for the first time, we find that miR-30b-5p may be a crucial regulator in the airway remodeling. This study offers some new clues to understand the molecular mechanism of asthma pathogenesis.

## 2. Materials and methods

### 2.1 Chemicals

Dulbecco’s modified Eagle’s medium (DMEM) was obtained from Hyclone (Logan, UT, USA); streptomycin, penicillin, PDGF, 3-(4,5-Dimethylthiazol-2-yl)-2,5-diphenyltetrazolium bromide (MTT), propidium iodide (PI) were obtained from Sigma-Aldrich (Louis, MO, USA); fetal bovine serum (FBS) was obtained from (Louis, MO, USA); The pcDNA3.1-PTEN (PTEN), control empty vector (control), miR-30b-5p inhibitors, miR-30b-5p mimics as well as their negative controls (NC) were bought from RiboBio (Guangzhou, China); Lipofectamine^TM^ 2000 was obtained from Invitrogen (Carlsbad, CA, USA). TRIzol reagent was obtained from Vazyme (Nanjing, China); PrimeScript RT Master Mix and SYBR PrimeScript™ miRNA RT-PCR kit were obtained from Takara (Dalian, China); the primers were provided by BGI (Shenzhen, China); 5-bromo-2-Deoxyuridine (BrdU) solution was obtained from BD Pharmingen (San Diego, CA, USA); RIPA lysis buffer was obtained from Beyotime (Shanghai, China); BCA protein detection kit was obtained from Pierce Chemicals Co. Ltd. (Rockford, IL, USA); the antibodies were obtained from Cell Signaling Technology (Danvers, MA, USA); enhanced chemiluminescence kit was obtained from Promega (Madison, WI, USA).

### 2.2 Cell culture

Human ASM cell line (ATCC; Manassas, VA, USA) was maintained in DMEM (Hyclone, Logan, UT, USA) with 0.1 mg/mL streptomycin and 100 U/mL penicillin (Sigma-Aldrich, Louis, MO, USA) as well as 10% FBS (Sigma-Aldrich, Louis, MO, USA). These cells were cultured in an incubator containing 5% CO_2_ in a humidified atmosphere at 37°C.

### 2.3 Cell transfection

The cells used in this study were transferred into 24-well plates (3 × 10^5^ cells/well), which were then cultured for 24 h at 37°C in 5% CO_2_. Subsequently, according to the manufacturer’s manual, Lipofectamine® 2000 (Invitrogen, Carlsbad, CA, USA) was adopted to perform the transfection. PDGF was used to induce the dysfunction of ASM cells [5,16]. 24 h after transfection, the cells were treated with 25 ng/mL PDGF (Sigma-Aldrich, Louis, MO, USA).

### 2.4 Quantitative real-time polymerase chain reaction (qRT-PCR) assay

The total RNA from ASM cells was extracted using TRIzol reagent (Vazyme, Nanjing, China), and the reverse transcription of mRNA and miRNA were conducted using PrimeScript RT Master Mix and SYBR PrimeScript™ miRNA RT-PCR kit (Takara, Dalian, China) based on the manufacturer’s instructions, respectively. qRT-PCR assay was conducted on CFX96 RT-PCR detection system (Bio-Rad, Hercules, CA, USA) and SYBR Premix Ex Taq (TaKaRa, Dalian, Japan). MiR-30b-5p and PTEN mRNA expressions were normalized to U6 and GAPDH, respectively. The specific primer sequences are as follows (F for forward; R for reverse): miR-30b-5p F, 5ʹ-ACGGGCAAAAATACTCCAGCTCTCAAT-3ʹ, miR-30b-5p R, 5ʹ-CTCTGGAAAACTGGTGTCGACTGGTGTC-3ʹ; U6 F, 5ʹ-CTCGCTTCGGCAGCACA-3ʹ, U6 R, 5ʹ-ACGCTTCACGAATTTGCGT-3ʹ; PTEN F, 5ʹ-AAGACCATAACCCACCACAGC-3ʹ, PTEN R, 5ʹ-ACCAGTTCGTCCCTTTCCAG-3ʹ; GAPDH F, 5ʹ-CAAAGGTGGATCAGATTCAAG-3ʹ, GAPDH R, 5ʹ-GGTGAGCATTATCACCCAGAA-3ʹ.

### 2.5 MTT assay

The transfected ASM cells were transferred into 96-well plates (3 × 10^4^ cells/well), cultured with the medium containing PDGF (25 ng/mL). After the cells were cultured at 37°C for 24 h, each well was added with 20 μL of MTT solution (5 mg/mL) (Sigma-Aldrich, Louis, MO, USA), and the cells were then incubated at 37°C for 6 h. Afterward, the medium was discarded, and MTT-formazan crystals were dissolved by dimethyl sulfoxide (150 μL/well) (DMSO; Sigma-Aldrich, Louis, MO, USA). An ELx808 absorbance microplate reader (Bio-Tek Instruments, Inc., Winooski, VT, USA) was utilized for measuring the absorbance at 570 nm.

### 2.6 BrdU assay

The transfected ASM cells were transferred to 96-well plates and cultured in the medium with PDGF (25 ng/mL) for 24 h. Subsequently, 10 μL of BrdU solution (BD Pharmingen, San Diego, CA, USA) was added to each well, and the cells were incubated for 4 h. Next, the cells were fixed and incubated with anti-BrdU antibody (Sigma-Aldrich, Louis, MO, USA) at room temperature for 1 h. After PBS washing three times, each well was supplemented with substrate solution (200 μL) and incubated for 25 min. Then, 25 μL of H_2_SO_4_ (1 mol/L) was added to terminate the reaction, and the well plates were shaken for 1 min. Each well was then added with 100 μL of Hoechst 33,342 staining solution for reaction for 30 min in the dark at room temperature. After PBS washing, the pictures were taken and the cells were counted under the fluorescence microscope.

### 2.7 Cell migration assay

Transwell chambers (24-well insert; pore size of 8 µm; Corning, NY, USA) were used to evaluate cell migration. ASM cells were re-suspended in serum-free DMEM. Next, the upper chamber was added with the cells (5 × 10^4^ cells/well), and the lower chamber was supplemented with DMEM (600 μL) with 10% FBS. After 24 h of incubation at room temperature, cotton swabs were utilized for removing the non-migrated cells remaining on the membrane’s upper surface, while the cells that had migrated to the bottom surface were fixed with 95% methanol and stained with 0.1% crystal violet. Pictures were taken and the migrated cells were counted in five randomly selected fields under the microscope.

### 2.8 Cell cycle assay

The transfected ASM cells were transferred into 6-well plates and treated with PDGF (25 ng/mL) for 24 h. After collection, the cells were fixed with ice-cold 70% (v/v) ethanol for 24 h. These cells were then washed with PBS, and these cells and RNase A (20 µg/mL) were incubated for 30 min at 37°C. Afterward, PI (100 µg/mL; Sigma-Aldrich, Louis, MO, USA) was employed to stain the cells for 10 min, and a FACScan flow cytometer (Becton-Dickinson, San Jose, CA, USA) was utilized for conducting flow cytometry analysis.

### 2.9 Dual-luciferase reporter assay

The mutant (MUT) and wild-type (WT) PTEN mRNA 3ʹUTR fragments containing miR-30b-5p binding sites were amplified by PCR and cloned into the pMIR-REPOR™ luciferase vector (Promega, Madison, WI, USA) to establish the luciferase reporter vector. The reporter vectors and miR-30b-5p mimics or control miRNAs were co-transfected into ASM cells with Lipofectamine 2000 (Invitrogen, Carlsbad, CA, USA). At 48 h after transfection, the cells were harvested, and a Dual-Luciferase Reporter Assay System (Promega, Madison, WI, USA) was utilized for measuring the luciferase activity. Renilla luciferase activity was used for normalization.

### 2.10 Western blotting

The total protein extraction from ASM cells was conducted using RIPA lysis buffer (Beyotime, Shanghai, China). A BCA protein detection kit (Pierce Chemicals Co, Rockford, IL, USA) was used for protein concentration measurement. Then equivalent amounts of protein (20 μg/lane) were dissolved by SDS-PAGE and then transferred to the PVDF membrane (Bio-Rad Laboratories Inc., Hercules, CA, USA). After the membrane was blocked with 5% skim milk for 1 h at room temperature, the membranes were incubated overnight with primary antibodies, including anti-phospho (p)-PI3K (1:1000, #17,366), anti-PI3K (1:1000, #4249), anti-PTEN (1:1000, #9188), anti-AKT (1:1000, #4691), anti-p-Akt (1:1000, #4060) and anti-GAPDH (1:1000, #5174) at 4°C GAPDH served as the endogenous control. After washing in tris buffered saline with tween (TBST), the membranes and horseradish peroxidase (HRP)-conjugated secondary antibody (1:1000, #7074, Cell Signaling Technology, Danvers, MA, USA) were incubated for 1 h at room temperature. Ultimately, an enhanced chemiluminescence kit (Promega, Madison, WI, USA) was adopted to visualize the protein bands, which were then analyzed by Image-Pro Plus software.

### 2.11 Statistical analysis

The statistical analysis tool was SPSS software (IBM, Armonk, NY, USA). All data from three independent replicates were expressed as ‘mean ± standard deviation’. One-way analysis of variance (ANOVA) (followed by Tukey’s post-hoc test) or Student’s *t*-test was conducted to calculate statistical differences. *P < *0.05 indicates statistically significance.

## 3. Results

MiR-30b-5p was screened out with bioinformatics analysis. To investigate the biological function of miR-30b-5p in regulating the phenotypes of ASM cells, PDGF was used to treat ASM cell, and then MTT, BrdU, Transwell and flow cytometry assays were performed. Additionally, the regulatory function of miR-30b-5p on PTEN was investigated by bioinformatics analysis and dual-luciferase reporter assay. Additionally, the regulatory effects of miR-30b-5p on PTEN, PI3K, p-PI3K, AKT and p-AKT expressions were investigated with Western blot. We demonstrated that, miR-30b-5p promoted the proliferation and migration of ASM cells induced by PDGF by repressing PTEN.

### 3.1. MiR-30b-5p is highly expressed in PDGF-induced human ASM cells

**Figure 1.**
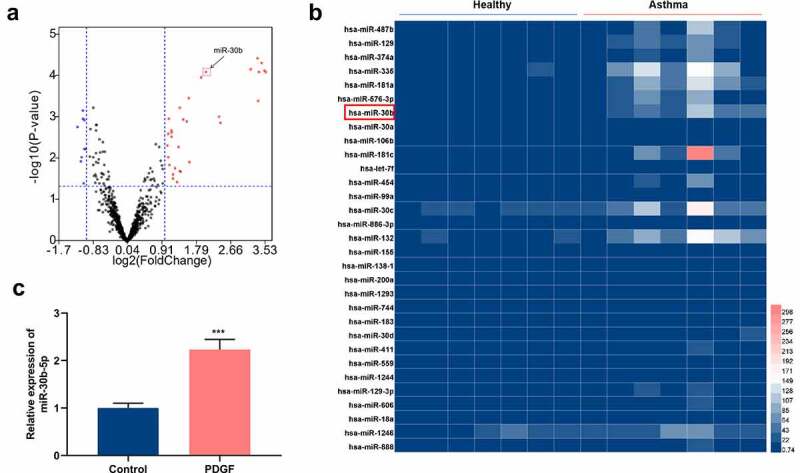
MiR-30b-5p is highly expressed in human ASM cells treated with PDGF

To unveil the roles of miRNAs in asthma, GSE25230 dataset was acquired from the gene expression omnibus (GEO) database (https://www.ncbi.nlm.nih.gov/). Through GEO2R analysis, a total of 41 differentially expressed miRNAs were identified. The data showed that in comparison with the healthy controls, 31 miRNAs were up-regulated in asthmatic bronchial epithelial cells, and 10 were down-regulated (Figure. 1a). Subsequently, miR-30b, whose expression was markedly up-regulated, was selected for the follow-up research (Figure 1b). To explore miR-30b-5p’s role in the pathogenesis of asthma, 25 ng/mL PDGF was used to treat ASM cells to construct an *in-vitro* cell model of asthma. qRT-PCR results showed that, as opposed to the control group, miR-30b-5p expression was dramatically enhanced in the ASM cells in the PDGF treatment group (Figure 1c). The aforementioned evidence revealed that miR-30b-5p might be implicated in asthma pathogenesis.

a. The volcano plot was used to show the expression changes of miRNAs in asthmatic bronchial epithelial cells. Horizontal axis: log2 (fold change); vertical axis: -log10 (*P*-value). The vertical line corresponds to 1.0 times up and down, and the horizontal line represents a *P*-value of 0.05. High expression is represented by ‘red’, low expression is represented by ‘blue’, and ‘black’ represents miRNAs with no significant differences. As shown, miR-30b-5p was among the up-regulated miRNAs in the bronchial epithelial cells obtained from asthma patients.b. The heat map was used to show 31 miRNAs, including miR-30b-5p, which were up-regulated in asthmatic bronchial epithelial cells.c. qRT-PCR was used to detect miR-30b-5p expression in human ASM cells with or without 25 ng/mL PDGF treatment. It showed that miR-30b-5p was markedly up-regulated in ASM cells after PDGF treatment.***P < 0.001.

### 3.2. MiR-30b-5p mediates the multiplication, migration and cell cycle progression of ASM cells induced by PDGF

**Figure 2.**
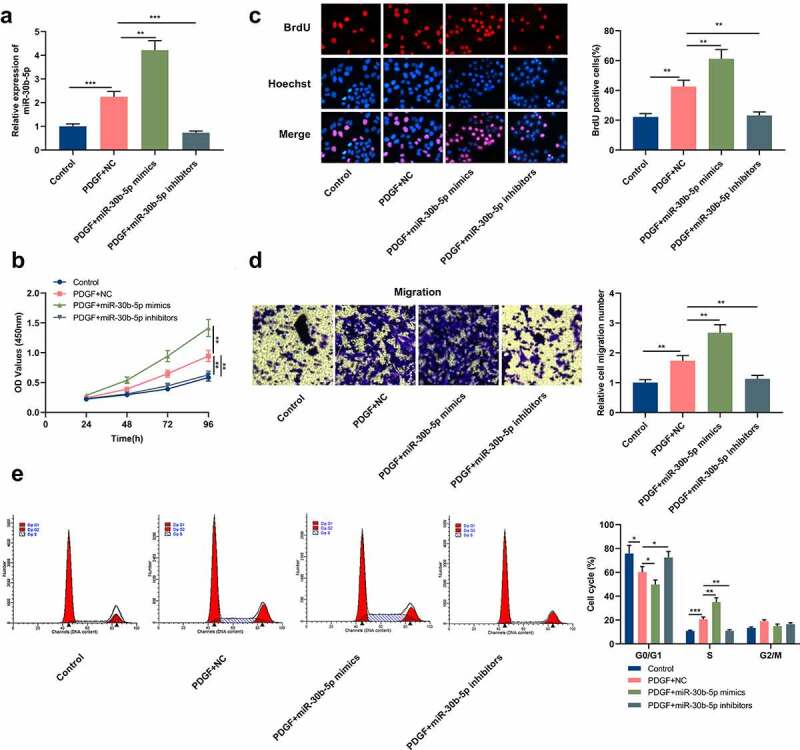
MiR-30b-5p promotes PDGF-induced human ASM cell proliferation, migration and cell cycle progression

To probe into miR-30b-5p’s biological functions in the pathogenesis of asthma, miR-30b-5p inhibitors or mimics were transfected into human ASM cells, and the transfection efficiency was verified through qRT-PCR. It was indicated that in comparison to the PDGF+NC group, miR-30b-5p expression was markedly elevated in the cells in the PDGF+miR-30b-5p mimic group, whereas miR-30b-5p expression was remarkably reduced in the cells in the PDGF+miR-30b-5p inhibitor group (Figure 2a). MTT, BrdU and Transwell assays were then conducted to detect cell multiplication and migration. It was found that as against the PDGF+NC group, the transfection of miR-30b-5p mimics markedly facilitated the multiplication and migration of PDGF-induced ASM cells, while miR-30b-5p inhibition restrained the multiplication and migration (Figure 2b-d). Furthermore, to clarify whether miR-30b-5p can repress cell multiplication by regulating cell cycle progression, flow cytometry was performed. It was revealed that as against the PDGF+NC group, the miR-30b-5p mimics transfection accelerated the G1 to S-phase cell cycle progression, whereas miR-30b-5p inhibition induced cell cycle arrest in G0/G1 phase (Figure 2e).

Human ASM cells were transfected with miR-30b-5p mimics or inhibitors, and then treated with 25 ng/mL PDGF for 24 h.a.MiR-30b-5p expression in ASM cells was detected by qRT-PCR, and it showed that the transfection was successful.b&c. ASM cells’ proliferation was detected by MTT and BrdU assays, and the results showed that miR-30b-5p positively regulated the proliferation of ASM cells.d.ASM cells’ migration was evaluated by Transwell assay, and the results showed that miR-30b-5p positively regulated the migration of ASM cells.e.ASM cells’ cell cycle was analyzed by flow cytometry, and the results showed that miR-30b-5p promoted the cell cell progression of ASM cells.**P < 0.01, and ***P < 0.001.

### 3.3 PTEN is the downstream target of miR-30b-5p in ASM cells

**Figure 3.**
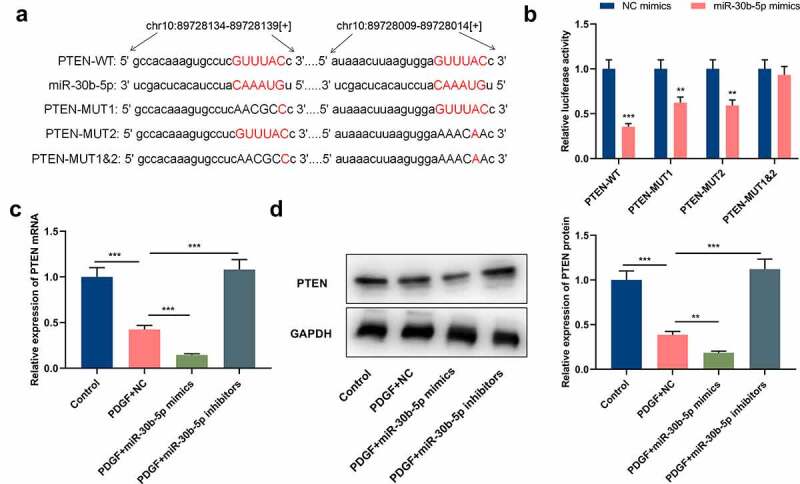
PTEN is a downstream target of miR-30b-5p

To clarify miR-30b-5p’s downstream mechanism in the pathogenesis of asthma, the StarBase database (http://starbase.sysu.edu.cn/) was utilized for predicting the downstream target genes of miR-30b-5p, and it was discovered that there were 2 complementary binding sites between PTEN mRNA 3ʹ-UTR and miR-30b-5p (Figure 3a). Dual-luciferase reporter gene assay manifested that as opposed to the NC group, the luciferase activities of PTEN-WT, PTEN-MUT1 and PTEN-MUT2 in the ASM cells of the miR-30b-5p mimic group were significantly reduced, whereas the luciferase activity of PTEN-MUT1&2 did not change significantly, which validated that the binding sites were functional (Figure 3b). qRT-PCR and Western blotting indicated that as opposed to the control group, PTEN mRNA and protein expressions were remarkably reduced in the PDGF treatment group (Figure 3c&d); compared with the PDGF+NC group, the transfection of miR-30b-5p mimics repressed PTEN mRNA and protein expressions, whereas miR-30b-5p inhibition induced the expression of PTEN (Figure 3c&d). Collectively, the aforementioned results suggested that miR-30b-5p directly targeted PTEN and inhibited its expression in ASM cells.

a. The binding sequences between miR-30b-5p and PTEN mRNA 3ʹ-UTR, which are predicted by bioinformatics analysis. b.The targeted relationship between miR-30b-5p and PTEN was verified by dual-luciferase reporter gene assay.c&d. qRT-PCR and Western blot were used to detect the expression of PTEN mRNA and protein in human ASM cells transfected with miR-30b-5p mimics and then treated with 25 ng/mL PDGF. The results showed that PDGF induced the down-regulation of PTEN expression in ASM cells, and miR-30b-5p negatively regulated PTEN expression.**P < 0.01, and ***P < 0.001.

### 3.4 PTEN restoration reverses the effect of miR-30b-5p on ASM cells treated with PDGF

**Figure 4.**
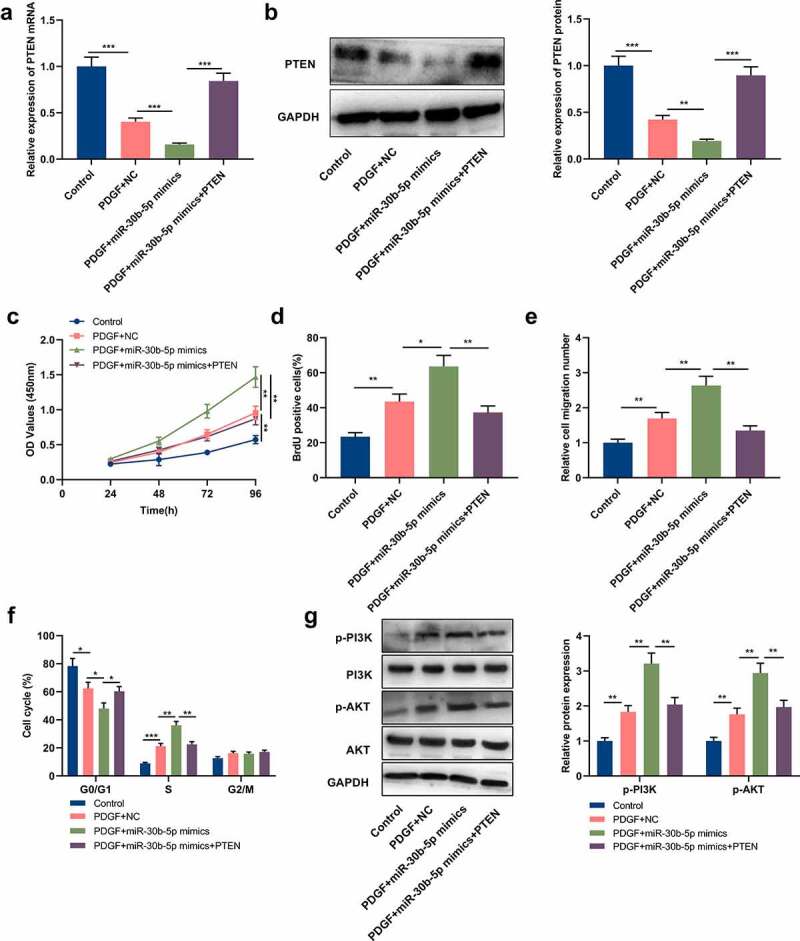
MiR-30b-5p participates in PDGF-mediated ASM cell proliferation and migration by regulating the PTEN/PI3K/AKT axis

To further clarify miR-30b-5p and PTEN’s biological functions in ASM cells, the cells were transfected with miR-30b-5p mimics and PTEN overexpression plasmids, respectively, or co-transfected with PTEN overexpression plasmids and miR-30b-5p mimics. PTEN expression in ASM cells was then detected by Western blot and qRT-PCR, and it was demonstrated that compared with the PDGF+miR-30b-5p mimics group, PTEN overexpression counteracted the inhibiting effect that the transfection of miR-30b-5p mimics had on PTEN mRNA and protein expressions (Figure 4a&b). Subsequently, MTT assay, BrdU assay, Transwell assay and flow cytometry analysis were conducted for detecting cell multiplication, migration and cycle progression, and it was unveiled that as opposed to the PDGF+miR-30b-5p mimics group, PTEN overexpression counteracted the promoting effects of miR-30b-5p overexpression on PDGF-induced ASM cell multiplication, migration and cycle progression (Figure 4c-f). Additionally, we also analyzed miR-30b-5p’s effect on the PTEN/PI3K/AKT signal pathway. Western blotting suggested that compared with the PDGF+NC group, p-AKT and p-PI3K protein expressions were notably increased in the cells of the PDGF+miR-30b-5p mimics group; in comparison with the PDGF+miR-30b-5p group, p-AKT and p-PI3K protein expressions were notably decreased in the PDGF+miR-30b-5p mimics+PTEN group (Figure 4g). The above-mentioned findings implied that miR-30b-5p could mediate PDGF-induced ASM cell multiplication, migration and cycle progression via targeting PTEN and modulating the PI3K/AKT signal pathway.

MiR-30b-5p mimics and PTEN overexpression plasmids were transfected into human ASM cells, or the cells were co-transfected with miR-30b-5p mimics and PTEN overexpression plasmids, and then the cells were stimulated with 25 ng/mL PDGF for 24 h.a&b. The relative expression of PTEN mRNA and protein in ASM cells were detected by qRT-PCR and Western blot.c&d. ASM cells’ proliferation was detected by MTT and BrdU assays. The results showed that PTEN restoration reversed the promoting effects of miR-30b-5p on ASM cells’ proliferation.e. Transwell assay was used to evaluate the migration of ASM cells. The results showed that PTEN restoration reversed the promoting effects of miR-30b-5p on ASM cells’ migration.f. Flow cytometry was used to analyze the cell cycle progression of ASM cells. The results showed that PTEN restoration reversed the promoting effects of miR-30b-5p on ASM cells’ cell cycle progression.g. Western blot was used to detect the expression of p-PI3K, p-AKT, PI3K and AKT proteins in ASM cells. The results showed that PDGF activated PI3K/AKT pathway, and miR-30b-5p further promoted the activation of PI3K/AKT pathway, and PTEN overexpression counteracted the effects of miR-30b-5p.**P < 0.01, and ***P < 0.001.

## 4. Discussion

Airway remodeling is a typical pathological feature of asthma, which can result in irreversible or partially irreversible airflow obstruction and bronchial hyperresponsiveness in asthma patients [17]. The increased ASM mass is a sign of airway remodeling, and it can lead to airway stenosis and obstruction [18]. It is known that the enhancement of ASM cell proliferation and migration contributes to the increase of ASM mass [4]. PDGF belongs to the growth factor family, which regulates mitosis and cell growth; it is secreted by asthmatic airway epithelial cells and inflammatory cells, and is mainly stored in α-granules of platelet [19]. PDGF can facilitate ASM cell multiplication and migration by activating multiple signal pathways (such as the PI3K/AKT, ERK), thereby aggravating airway remodeling in asthma [20,21]. In the present work, PDGF was used to treat ASM cells to construct the in vitro model of asthma, and we demonstrated that PDGF treatment facilitated the growth and migration of ASM cells, which is consistent with the previous reports [5,16,20,21].

MiRNAs can modulate various cellular processes, for instance, cell differentiation, proliferation, apoptosis, migration and autophagy, and they are related to the pathogenesis of asthma and many other human diseases [22,23]. Specifically, miR-192-5p is down-regulated in the lung tissues of asthmatic mice, and it can alleviate asthmatic airway remodeling and autophagy via targeting ATG7 and MMP-16 [24]. High miR-126 expression is found in the asthmatic mice’s lung tissues and in the serum exosomes of allergic asthma patients [25]. miR-375 suppresses PDGF-induced ASM cell multiplication and migration via modulating the JAK2/STAT3 signal transduction [26]; miR-638 restrains PDGF-BB-induced human ASM cell multiplication and migration by targeting cyclin D1 and NOR1 [27]. The current study validated that miR-30b-5p was a new regulator of ASM cells’ multiplication and migration. MiR-30b-5p is reportedly down-regulated in multiple tumors, including esophageal squamous cell carcinoma, glioma, and hepatocellular carcinoma, and participates in inhibiting the progression of tumors [9–11]. In this work, it was demonstrated that miR-30b-5p was highly expressed in asthmatic bronchial epithelial cells. Additionally, PDGF treatment promoted miR-30b-5p expression in ASM cells. Furthermore, it was discovered that miR-30b-5p facilitated PDGF-induced ASM cell multiplication, migration and cycle progression, whereas miR-30b-5p inhibition exerted the opposite effect. The aforementioned evidence revealed that miR-30b-5p might be a crucial regulator for ASM cell multiplication and migration in asthmatic airway remodeling. To our best knowledge, for the first time, miR-30b-5p is reported to be associated with asthma pathogenesis, suggesting it may function as a therapy target for asthma.

PTEN protein has dual-specificity phosphatase activity; it is not only pivotal in modulating embryonic development, cell growth, differentiation, apoptosis and migration, but also regulates cell signaling pathways such as PI3K/AKT, mitogen-activated protein kinase (MAPK) and focal adhesion kinase (FAK) to mediate the occurrence and development of diseases [28–30]. PTEN is reportedly down-regulated in the ASM cells of mice with allergic asthma induced by ovalbumin [15]. PTEN can inhibit asthmatic airway remodeling by regulating the CD38-mediated Ca^2+^/CREB signal pathway [31]. PTEN negatively regulates the PI3K/AKT signal pathway, and plays a role mainly through the dephosphorylation of PIP3 [14]. There is emerging evidence indicating that activating the PI3K/AKT pathway promotes the dysfunction of ASM cell [32,33]. Additionally, some studies report that miRNAs take part in the pathogenesis of asthma via regulating PTEN/PI3K/AKT pathway. For example, the transfection of miR-21 mimics inhibits PI3K/AKT signaling via targeting PTEN, thereby enhancing the sensitivity of ASM cells to glucocorticoids [34]; miR-19a promotes human ASM cell proliferation and migration induced by high-mobility group protein B1 through modulating the PTEN/AKT pathway [35]. In the current study, PTEN was identified as the target gene of miR-30b-5p, and miR-30b-5p negatively regulated PTEN mRNA and protein expression in PDGF-induced ASM cells. Furthermore, it was discovered that the promoting impact of miR-30b-5p mimics transfection on PDGF-induced ASM cell multiplication, migration and cell cycle could be weakened by PTEN overexpression. Moreover, the transfection of miR-30b-5p mimics could activate the PI3K/AKT signaling pathway, while PTEN overexpression could attenuate this effect. The above findings revealed that miR-30b-5p could participate in PDGF-induced ASM cell multiplication, migration and cycle progression via regulating the PTEN/PI3K/AKT signal pathway. These findings partly explained the mechanism of the dysregulation of PTEN/PI3K/AKT signal pathway in ASM cells.

## 5. Conclusion

To sum up, the present work shows that miR-30b-5p mediates the multiplication, migration and cycle progression of PDGF-induced human ASM cells, through regulating PTEN/PI3K/AKT signal pathway. Our findings imply that targeting miR-30b-5p/PTEN axis in human ASM cells may offer a new treatment option for the prevention of airway remodeling in asthma. Nonetheless, only *in vitro* experiments were performed in the present work, and in the future, animal models are required to further validate our demonstrations.

## Data Availability

The data used to support the findings of this study are available from the corresponding author upon request.

## Highlights

1. **The expression level of miR-30b-5p is reduced in airway smooth muscle cells with platelet-derived growth factor stimulation.**
2. **MiR-30b-5p represses the growth and migration of airway smooth muscle cells, which contributes to regulating airway remodeling in the pathogenesis of asthma.**
3. **phosphatase and tensin homolog deleted on chromosome ten is a target gene of miR-30b-5p in airway smooth muscle cells.**

## List of abbreviations

MiR-30b-5p; microRNA-30b-5p: PTEN; phosphatase and tensin homolog deleted on chromosome ten: ASM, airway smooth muscle; PDGF; platelet-derived growth factor: DMEM; Dulbecco’s modified Eagle’s medium: qRT-PCR; quantitative real-time polymerase chain reaction: TBST; tris buffered saline with tween: PI3K; phosphatidylinositol 3-kinase: AKT; protein kinase B: 3ʹUTR; 3ʹ-untranslated region: MTT; 3-(4,5-Dimethylthiazol-2-yl)-2,5-diphenyltetrazolium bromide: BrdU; 5-bromo-2ʹ-deoxyuridine: PI; propidium iodide: GEO; gene expression omnibus
